# Functional nutritional frailty predicts complications after microvascular free flap reconstruction

**DOI:** 10.3389/fsurg.2026.1915384

**Published:** 2026-08-12

**Authors:** Çağla Çiçek, Samed O. Akın, Cem Aydın, Gaye Filinte

**Affiliations:** 1 Department of Plastic, Reconstructive and Aesthetic Surgery, Kartal Dr. Lütfi Kırdar City Hospital, Istanbul, Türkiye; 2 Department of Plastic, Reconstructive and Aesthetic Surgery, University of Health Sciences, Kartal Dr. Lütfi Kırdar City Hospital, Istanbul, Türkiye; 3 Private Practitioner, Istanbul, Türkiye

**Keywords:** frailty, free tissue transfer, malnutrition, nutritional screening, postoperative complications, sarcopenia

## Abstract

**Background:**

Outcomes after microvascular free flap reconstruction depend not only on surgical technique but also on patient-related factors such as nutritional and functional reserve. However, conventional biochemical markers may not fully reflect the patient's physiological reserve. This study aimed to evaluate whether functional nutritional screening tools can predict free flap–related complications.

**Methods:**

Eighty patients who underwent microvascular free flap reconstruction were retrospectively analyzed. Preoperative nutritional status was assessed using the Mini Nutritional Assessment (MNA) and SARC-F questionnaire. Patients were categorized as high risk (HR) or low risk (LR) according to these screening tools. Free flap–related complications within 30 days were compared between groups. Multivariable logistic regression analysis was performed as an exploratory analysis to evaluate the association between functional nutritional frailty and flap-related complications.

**Results:**

Twenty-nine patients (36.3%) were classified as high risk. Flap-related complications, defined as a composite outcome including total flap loss, partial flap necrosis, vascular thrombosis requiring re-exploration, surgical site infection, and wound dehiscence, occurred in 17 patients (21.3%). Complication rates were significantly higher in the HR group compared with the LR group (55.2% vs. 1.9%, *p* < 0.001), corresponding to an approximately 29-fold increase in risk. In multivariable analysis, functional nutritional frailty remained associated with postoperative complications in the exploratory multivariable model (OR 74.4, 95% CI 8.8–629.6, *p* < 0.001), whereas diabetes mellitus and smoking were not associated with complication development.

**Conclusion:**

Functional nutritional frailty assessed by simple clinical screening tools such as MNA and SARC-F was strongly associated with flap-related complications after free flap reconstruction. Incorporating these tools into routine preoperative assessment may help identify high-risk patients and optimize perioperative management.

## Introduction

Free tissue transfer is one of the most advanced techniques in plastic and reconstructive surgery and provides functionally and aesthetically successful outcomes in the reconstruction of large and complex tissue defects (1). With the evolution of surgical techniques, increased microsurgical experience, and improved perioperative care, success rates for free flap surgery have risen substantially. However, patient-related factors that influence flap survival and surgical outcomes remain an important area of investigation for clinicians. The patient's nutritional status plays a decisive role in wound healing, infection risk, and the postoperative recovery process following major surgical procedures (2). It is well established that malnutrition is associated with increased morbidity and mortality and elevates the risk of surgical complications (3). In operations that impose high physiological stress—such as free tissue transfer—adequate nutritional reserves are critical for tissue repair and flap viability. In clinical practice, nutritional status is most often assessed using body mass index (BMI) and biochemical parameters such as serum albumin and prealbumin. However, these measures can fail to detect occult malnutrition, particularly in patients with normal BMI values (4). In patients with reduced muscle mass or diminished functional capacity, classical anthropometric and biochemical indices may not always reflect true physiological reserve (5). In recent years, emphasis has shifted toward assessing nutritional status not only by laboratory values but also by patients' functional capacity and musculoskeletal condition. Screening instruments that are easy to apply in clinical practice—such as the Mini Nutritional Assessment (MNA) and the SARC-F questionnaire—have gained increasing importance for identifying sarcopenia and occult malnutrition risk (6, 7) (Table 1). Nevertheless, the use of functional nutritional screening tools to assess nutritional status and their relationship with flap outcomes in patients undergoing free tissue transfer has been only sparsely investigated in the literature (8, 9). In particular, the role of practical clinical screening tools such as the MNA and SARC-F in predicting complications after free flap surgery has not been sufficiently explored. The aim of this study was to investigate the impact of occult malnutrition and sarcopenia risk—assessed by the MNA and SARC-F scores in addition to conventional nutritional evaluation methods—on flap viability and postoperative complications in patients undergoing free tissue transfer.

**Table 1:** Nutritional frailty screening.

Screening tool and classification thresholds
Mini nutritional assessment
• Score range: 0–30
• <17 → Malnutrition
• 17–23.5 → Risk of malnutrition
• 24–30 → Normal nutritional status
SARC-F Questionnaire
• Score range: 0–10
• ≥4 → Risk of sarcopenia
• <4 → No sarcopenia risk

Classification thresholds for the MNA and SARC-F scoring systems.

## Materials and methods

**Figure 1:**
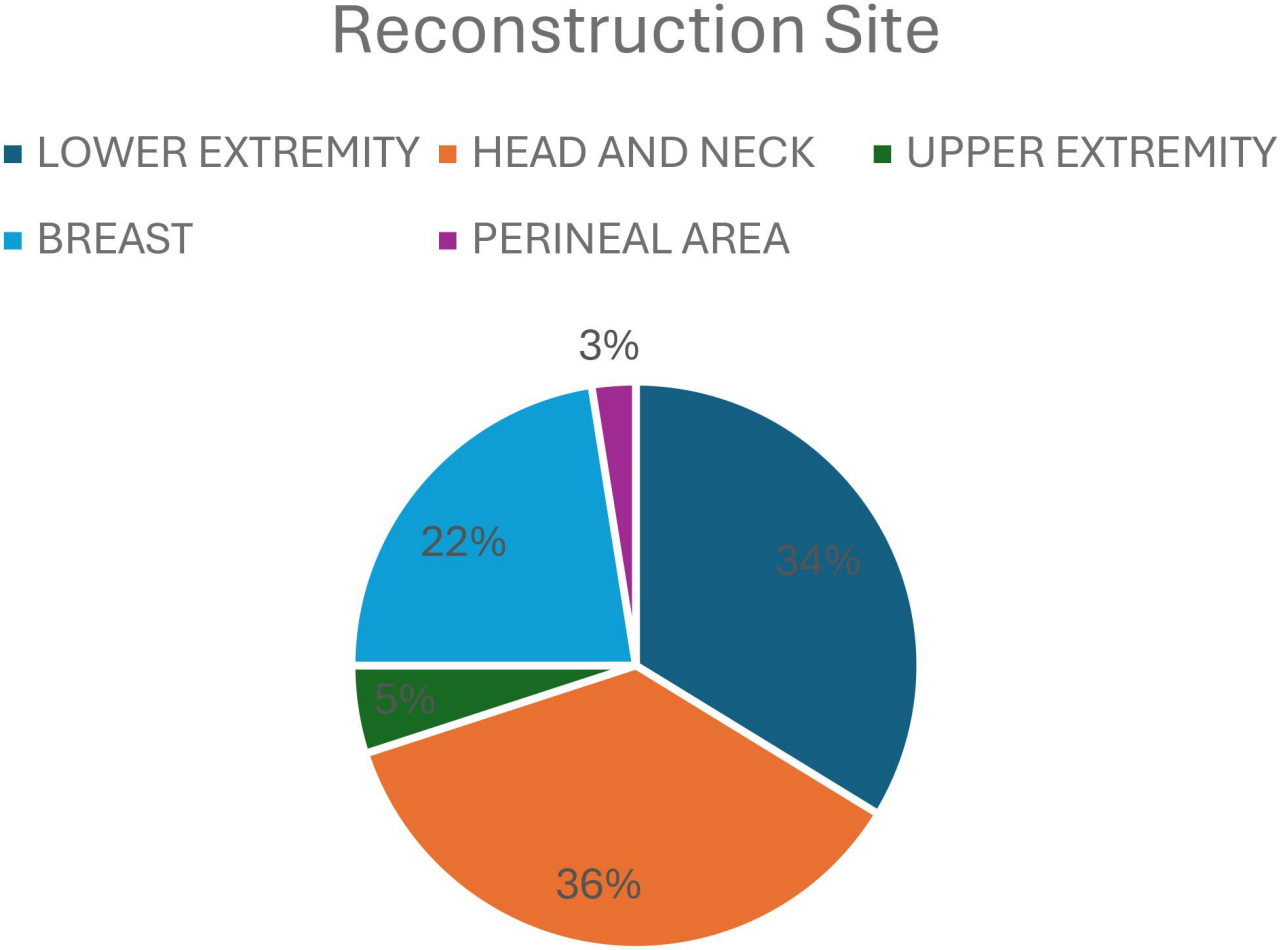
Reconstruction site. Distribution of reconstruction sites: of 80 patients, 27 underwent lower-extremity reconstruction, 29 underwent head-and-neck reconstruction, 18 underwent breast reconstruction, 4 underwent upper-extremity reconstruction, and 2 underwent perineal reconstruction.

**Figure 2:**
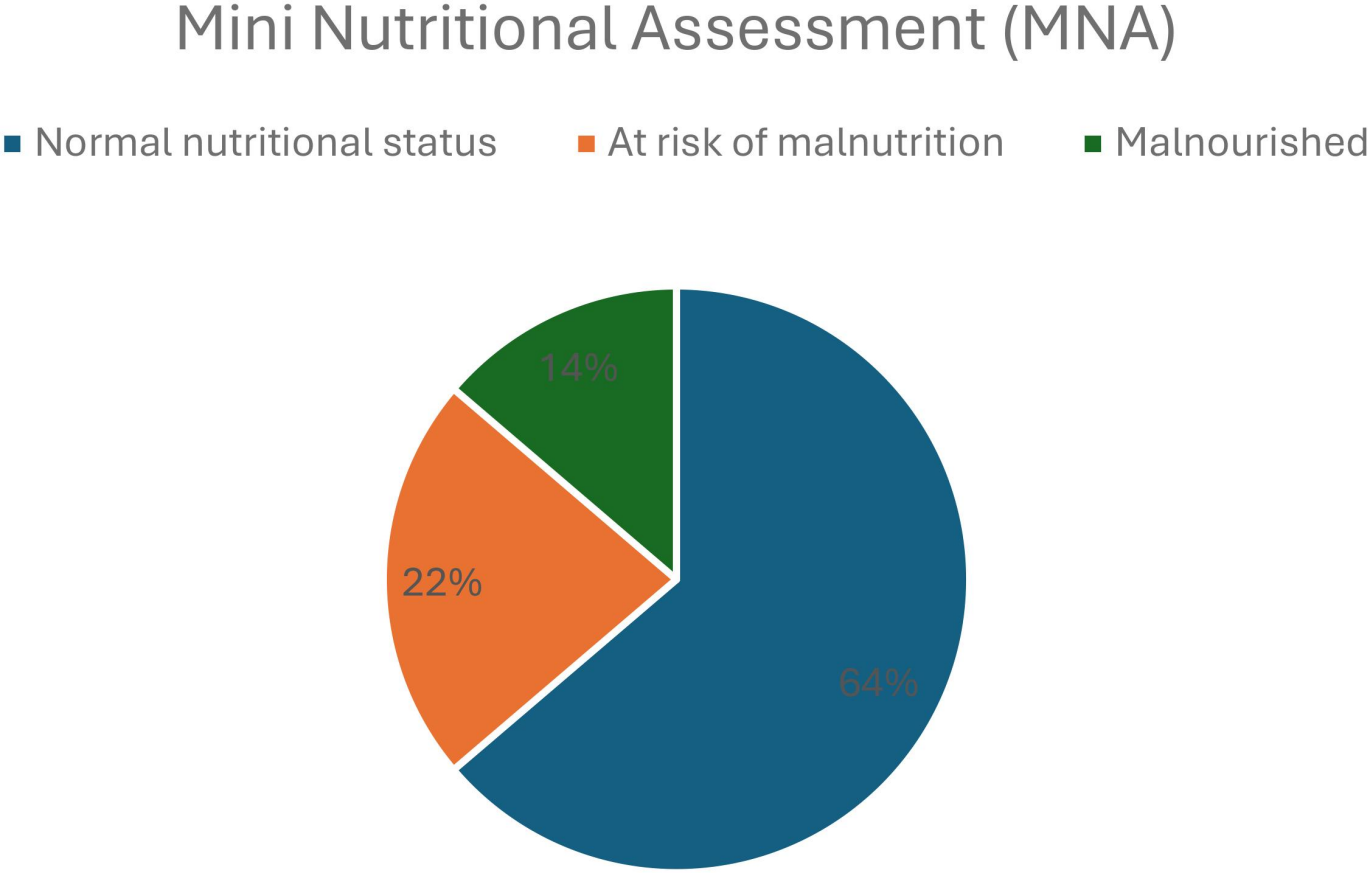
Mini nutritional assessment (MNA). Following MNA scoring, 51 of 80 patients were classified as having normal nutritional status, 18 were classified as at risk of malnutrition, and 11 were classified as malnourished.

This investigation was designed as a single-center, retrospective observational cohort study to evaluate the effect of nutritional status on flap viability and postoperative complications in patients who underwent free tissue transfer at the Kartal Dr. Lütfi Kırdar City Hospital, Plastic Reconstructive and Aesthetic Surgery Clinic. Approval for the study was obtained from the local ethics committee (approval number 2025/010.99/12/32). Eighty patients who underwent free tissue transfer at our clinic between January 2024 and December 2024 and for whom complete hospital medical records were available were included in the study. Patients aged 18 years and older were eligible for inclusion; cases with missing preoperative nutritional assessment data (Mini Nutritional Assessment or SARC-F score) or incomplete clinical follow-up were excluded. The Mini Nutritional Assessment (MNA) evaluates nutritional status using items related to anthropometric measurements, recent weight loss, dietary intake, mobility, psychological stress or acute illness, neuropsychological status, and self-perceived health and nutrition. Total scores range from 0 to 30, with scores ≥24 indicating normal nutritional status, 17–23.5 indicating risk of malnutrition, and <17 indicating malnutrition. The SARC-F questionnaire is a five-item screening tool assessing strength, assistance with walking, rising from a chair, climbing stairs, and falls. Scores range from 0 to 10, with a score of ≥4 indicating probable sarcopenia and impaired functional reserve. All free tissue transfers were performed by the same surgical team. To minimize potential bias, strict inclusion and exclusion criteria were uniformly applied to prevent selection bias. To mitigate information and measurement bias, all biochemical analyses were performed in a single central laboratory using standardized equipment, and clinical data were cross-checked. Additionally, the questionnaire was administered by trained researchers using a standardized protocol with clear, non-leading questions to eliminate interviewer and recall bias. The cases included free flap reconstructions performed for indications in the head and neck region, the extremities, and breast reconstruction. Flap type, defect location, total operative time, and flap ischemia time were reviewed retrospectively from existing records (Figure 1). Postoperatively, all free flaps were monitored by clinical examination and handheld Doppler hourly for the first 24 h and then at decreasing frequency thereafter. The anastomotic line was routinely assessed. Daily postoperative complete blood counts and acute-phase reactants were recorded from the patient files and evaluated. Antibiotic administration was planned according to patient and surgical characteristics rather than a standardized protocol. Preoperative nutritional status was also assessed retrospectively using records in the patient files. Anthropometric and laboratory data recorded for the study included BMI; serum albumin and prealbumin (transthyretin) levels; hemoglobin; blood urea and creatinine; total lymphocyte count; neutrophil-to-lymphocyte ratio to better characterize inflammatory status; and preoperative HbA1c in patients with a diagnosis of diabetes mellitus to assess metabolic control. In addition, within the first 72 postoperative hours it was recorded whether patients required packed red blood cell (PRBC) transfusion, whether they were active smokers, and whether they had experienced ≥5% weight loss in the preceding six months. The Mini Nutritional Assessment (MNA) and the SARC-F score were used as clinical screening tests (Figure 2). The combined use of these two screening instruments allows simultaneous evaluation of nutritional status and muscle strength/functional capacity; thus, patients were assessed not only by biochemical parameters but also by functional nutritional status. MNA and SARC-F evaluate different but complementary physiological domains, and impairments identified by either test may reflect reduced physiological reserve in the face of surgical stress and functionally relevant, functional nutritional frailty. For this reason, patients with a positive result on either test were classified as being in the risk group. Accordingly, patients were divided into two groups: those with malnutrition or risk of malnutrition according to the MNA and/or SARC-F score ≥4 — the High-Risk Group (HR) (*n* = 29) — and those with normal nutritional status by MNA and without sarcopenia risk by SARC-F — the Low-Risk Group (LR) (*n* = 51). MNA and SARC-F were used in combination because they assess complementary dimensions of patient vulnerability. While the MNA evaluates nutritional status, SARC-F reflects functional performance and sarcopenia-related reserve. Their combined assessment was intended to identify patients with nutritional and/or functional vulnerability, representing the broader concept of functional nutritional frailty. This grouping aimed to evaluate the effect on flap outcomes of patients who may carry occult malnutrition and sarcopenia risk despite having BMI values within normal limits. The primary outcome of the study was the occurrence of flap-related complications within the first 30 postoperative days. Flap-related complications were defined as a composite outcome including total flap loss, partial flap necrosis, arterial or venous thrombosis requiring surgical re-exploration, flap salvage procedures or surgical revision for vascular compromise, surgical site infection, and wound dehiscence. Length of hospital stay and hospital readmission within 30 days were recorded separately as additional postoperative outcomes.

### Statistical analysis

A formal power analysis was not performed; instead, a time-frame based sampling approach was used. The study size was determined by including all patients who met the inclusion criteria and visited the clinic between January 2024 and December 2024. During this period, a total of 80 eligible participants were identified and included in the final analysis. Statistical analyses were performed using IBM SPSS Statistics for Windows, version 29.0.2.0 (IBM Corp., Armonk, NY, USA). Continuous variables were presented as mean ± standard deviation or median (minimum-maximum). Categorical variables were expressed as numbers and percentages. The HR and LR groups were compared with respect to clinical outcomes. Variables with missing data were excluded from analyses. Continuous variables were compared using Student's t-test and presented as mean ± SD. Categorical variables were analyzed with Fisher's exact test. Multivariable logistic regression analysis was performed as an exploratory analysis to evaluate the association between functional nutritional frailty and flap-related complications. Results were reported as odds ratios (ORs) with 95% confidence intervals (CIs). A *p* value <0.05 was considered statistically significant in all analyses.

## Results

A total of 80 patients who underwent free tissue transfer were included in the study. The mean patient age was 50.5 ± 15.1 years (range 19–87) and mean BMI was 24.01 ± 5.4 kg/m^2^ (range 13.1–34.5). Mean serum albumin was 3.74 ± 0.92 g/dL and mean prealbumin was 0.20 ± 0.04 g/dL. Mean hemoglobin level was 12.5 (9.4−15.3) g/dL. Mean neutrophil-to-lymphocyte ratio (NLR) was 2.38 ± 0.69, and the mean SARC-F score was 1.43 (Table 2). According to MNA and/or SARC-F assessment, 29 patients (36.3%) comprised the High-Risk (HR) group, while 51 patients (63.7%) were classified as nutritionally normal (Low-Risk, LR). Additional analyses were performed to evaluate the contribution of the individual screening tools. Patients classified as being at nutritional risk or malnourished according to the MNA had a significantly higher postoperative complication rate than those with normal nutritional status [55.2% (16/29) vs. 2.0% (1/51)]. Likewise, patients with a SARC-F score ≥4 had a substantially higher complication rate than those with a score <4 [92.3% (12/13) vs. 7.5% (5/67)]. When the two instruments were considered together, postoperative complications occurred in 2.0% (1/51) of patients with neither nutritional nor functional impairment, 25.0% (4/16) of patients with nutritional risk alone, and 92.3% (12/13) of patients with both nutritional risk and impaired functional status. Fifteen patients (18.0%) were active smokers. Four patients (5.0%) had a diagnosis of diabetes mellitus based on HbA1c measurements. Within the first 72 postoperative hours, 37 patients (46.25%) required packed red blood cell transfusion. The 30-day readmission rate was 28.8%. Thirty-day readmissions were mainly related to wound-healing problems, localized surgical site infections requiring intravenous antibiotics, partial necrosis requiring local wound care, and donor-site morbidity, rather than late total flap failure. Given that our cohort included highly complex reconstructions (such as head and neck and lower extremity cases) and a substantial proportion of patients with high functional nutritional risk, this rate reflects the close post-operative monitoring required for early wound and infectious complications rather than catastrophic flap failures. Managing these minor complications through short-term readmissions remains a critical component of ensuring long-term reconstructive success in fragile patients. Within the first 30 postoperative days, flap-related complications occurred in 17 patients (21.3%) (Table 3). The complication rate in the HR group was 55.2% (16/29), whereas it was 1.9% (1/51) in the LR group. The difference between groups was statistically significant (Fisher's exact test, *p* < 0.001). Accordingly, the risk ratio between groups was calculated to be approximately 29. Multivariate logistic regression analysis to evaluate the independent effect of functional nutritional frailty included malnutrition status, diabetes mellitus, and smoking. In the exploratory multivariable logistic regression model, functional nutritional frailty remained associated with flap-related complications (OR 74.4, 95% CI 8.8–629.6, *p* < 0.001), whereas diabetes mellitus and smoking were not significantly associated with complication development (Table 4). Biochemical and anthropometric parameters were compared between patients who did and did not develop flap-related complications. Patients who developed complications had significantly lower mean serum albumin levels (2.49 ± 0.54 g/dL vs. 4.08 ± 0.58 g/dL; *p* < 0.001). Prealbumin levels were also lower in the complication group (0.14 ± 0.02 g/dL vs. 0.22 ± 0.03 g/dL; *p* < 0.001). When the relationship between BMI and complications was evaluated, patients with complications had a lower BMI (17.8 ± 3.1 kg/m^2^ vs. 25.7 ± 4.2 kg/m^2^; *p* < 0.001). Hemoglobin levels were likewise lower in the complication group (9.98 ± 0.87 g/dL vs. 13.24 ± 1.12 g/dL; *p* < 0.001). Although NLR tended to be higher in the complication group, this difference did not reach statistical significance (*p* > 0.05). By contrast, patients classified as HR by MNA and/or SARC-F had significantly higher NLR values compared with the LR group (2.81 ± 0.72 and 2.13 ± 0.53, *p* < 0.001) (5, 6). This finding suggests higher baseline systemic inflammation in patients with nutritional and functional frailty. Patients who required transfusion within the first 72 postoperative hours showed a higher rate of flap-related complications; however, this association was not statistically significant (*p* > 0.05). Overall, these results indicate that conventional biochemical markers of malnutrition were associated with the development of flap-related complications. In the exploratory multivariable model, high-risk status defined by MNA and/or SARC-F remained associated with flap-related complications.

**Table 2:** Baseline demographic, nutritional, and laboratory characteristics of the study population (*n* = 80).

Variable	Value
Age, years	50.6 ± 15.1
BMI, kg/m²	24.0 ± 5.4
Hemoglobin, g/dL	12.5 ± 2.1
Total lymphocyte count	1.98 ± 0.96
Albumin, g/dL	3.74 ± 0.92
Prealbumin, g/dL	0.20 ± 0.04
Urea, mg/dL	24.8 ± 8.0
Creatinine, mg/dL	1.10 ± 0.38
SARC-F score	1.44 ± 1.83
Diabetes mellitus, *n* (%)	4 (5.0)
Active smoking, *n* (%)	15 (18.8)
Flap-related complication, *n* (%)	17 (21.3)
Rehospitalization, *n* (%)	23 (28.8)
High-risk nutritional frailty (MNA risk/malnutrition and/or SARC-F ≥4), *n* (%)	29 (36.3)
Low-risk nutritional frailty, *n* (%)	51 (63.7)

Continuous variables are presented as mean ± standard deviation and categorical variables as number (percentage). High-risk nutritional frailty was defined as the presence of malnutrition/risk of malnutrition on MNA and/or SARC-F score ≥4.

**Table 3:** Distribution of the components of the composite flap-related complication outcome within the first 30 postoperative days. The primary outcome was defined as a composite of flap-related complications, including total flap loss, partial flap necrosis, arterial or venous thrombosis requiring surgical re-exploration, flap salvage/revision surgery, surgical site infection, and wound dehiscence. Because individual patients could experience more than one complication, the total number of complication events may exceed the number of patients with complications.

Complication	*n*	%
Total flap loss	3	3.8
Partial necrosis	4	5.0
Venous thrombosis	5	6.3
Arterial thrombosis	2	2.5
Revision surgery	7	8.8
SSI	6	7.5
Wound dehiscence	4	5.0

**Table 4:** Multivariable logistic regression analysis for flap-related complications.

Variable	Odds ratio (OR)	95% CI	*p*-value
High-risk nutritional frailty (MNA/SARC-F based)	74.4	8.8–629.6	<0.001
Diabetes mellitus	0.23	0.02–2.65	0.240
Active smoking	1.20	0.20–7.03	0.841

In the exploratory multivariable logistic regression model, functional nutritional frailty defined by MNA and/or SARC-F remained associated with flap-related complications. Owing to the limited sample size and small number of events, these findings should be interpreted cautiously.

## Discussion

Although free tissue transfer offers high success rates for the reconstruction of large tissue defects, flap viability and postoperative complications remain important clinical challenges (10). Therefore, identification of patient-related risk factors that affect surgical success is of great importance for both patient selection and perioperative management. Reported complication rates after microvascular free flap surgery vary across series and are approximately 10%–30% (11). Advanced age, comorbid disease, tobacco use, operative duration, and factors affecting systemic perfusion have been identified in the literature as potential risk factors for flap failure (12). However, in recent years it has been increasingly emphasized that surgical outcomes may be influenced not only by technical factors but also by the patient's physiological reserve and frailty (13). In the present study, assessing nutritional status with screening tools that incorporate functional capacity in addition to conventional biochemical parameters demonstrated an important role in predicting flap-related complications. The most striking finding of the study is the strong association between nutrition-related functional risk—defined by the MNA and/or SARC-F—and the development of complications after free flap surgery. The complication rate in the high-risk group was 55.2%, whereas it was only 1.9% in the nutritionally low-risk group, corresponding to an approximately 29-fold increase in complication risk (Table 5). Moreover, in multivariate analysis the functional nutritional frailty remained associated with postoperative complications in the exploratory multivariable model, while traditional risk factors such as diabetes mellitus and smoking lost statistical significance. Although diabetes mellitus and smoking were not significantly associated with postoperative complications in the present multivariable analysis, these findings should not be interpreted as evidence that these factors are clinically unimportant. Given the limited sample size and number of events, the study may have been underpowered to detect such associations. Larger studies are needed to further evaluate their potential contribution to postoperative outcomes. It has long been recognized that malnutrition is associated with increased morbidity and mortality after major surgical procedures. Mechanisms underlying this association include inability to meet increased metabolic demands during surgical stress, impaired immune response, and delayed wound healing (5, 14). Studies in the field of reconstructive surgery have likewise shown that preoperative nutritional status may substantially affect outcomes of free flap surgery. In a multicenter database study, Yen et al. reported an association between poor nutritional indicators and flap failure (15). Similarly, Herzog et al. demonstrated that malnutrition increased the risk of wound complications and flap failure in patients undergoing head and neck reconstruction (16). In our cohort, the significantly lower serum albumin and prealbumin levels observed in patients who developed complications are concordant with these reports. Although classical nutritional parameters such as albumin, prealbumin, and BMI were significantly lower in patients with complications in the present study, they did not remain independent predictors in the multivariable analysis. Although the mean BMI in the high-risk group remained close to the lower limit of the normal range, BMI alone is an imperfect marker of nutritional status because it does not reflect muscle mass, functional capacity, recent nutritional decline, or sarcopenia-related reserve. This finding further supports the use of multidimensional assessment tools such as MNA and SARC-F, which provide a more comprehensive evaluation of functional nutritional frailty. In our study, traditional biochemical markers such as albumin, prealbumin, and hemoglobin, along with BMI, were significantly lower in patients who developed postoperative complications. While these markers are valuable clinical indicators, they represent isolated biological parameters that can fluctuate due to acute phase responses, fluid shifts, and systemic inflammation. Consequently, they may not fully reflect a patient's actual homeostatic capacity. By contrast, the nutrition-related functional risk state identified by MNA and SARC-F showed the strongest association with complications in the present cohort. This observation suggests that surgical outcomes may be more closely related to the patient's functional physiological reserve than to isolated biochemical markers. Serum albumin and prealbumin have been used for many years in nutritional assessment; prealbumin, in particular, has a short half-life and may reflect changes in nutritional status more rapidly, and some studies have reported low prealbumin as a risk factor for free flap failure (17). Nevertheless, these biomarkers have important limitations: albumin concentration is influenced by inflammation, acute-phase responses, and fluid balance (18). Therefore, nutritional assessment based solely on biochemical markers may not fully reflect true physiological reserve. This limitation can be understood in the context of the growing recognition of sarcopenia and functional frailty in the surgical literature. Sarcopenia—characterized by progressive loss of muscle mass and strength—is associated with diminished physiological reserve in the face of surgical stress (19). Studies in reconstructive surgery have shown that sarcopenia may increase the risk of complications after free flap procedures (8). However, most of those studies rely on imaging-based measures of muscle area (e.g., computed tomography). The distinctive aspect of the present study is that nutritional reserve was evaluated not only by biochemical parameters or imaging-based muscle metrics but also by simple, clinically applicable functional screening tools. Tests such as the MNA and SARC-F allow rapid, practical assessment of nutritional status and functional capacity. The combined use of these instruments may provide a more holistic reflection of the patient's physiological reserve against surgical stress (20, 21). These findings point to an important clinical reality: patients may have reduced physiological reserve despite normal laboratory values. In particular, patients with a normal BMI may nonetheless have muscle mass loss and impaired functional capacity. The concept of the “normal-weight malnourished” patient is increasingly recognized (22). Hence, assessments based solely on BMI or serum protein levels may fail to detect true nutritional risk. A clinically relevant implication of the present study is that simple preoperative screening tests may be useful for risk stratification prior to free flap surgery. Administration of instruments such as the MNA and SARC-F requires only a few minutes and incurs no additional cost. Therefore, incorporating these tests into routine preoperative evaluation could facilitate early identification of high-risk patients. In conclusion, this study demonstrates that nutrition-related functional frailty is a strong predictor of flap-related complications in patients undergoing free tissue transfer. A clinically important implication of these findings is that nutrition-related functional frailty can be identified early in the preoperative period in patients planned for free flap surgery. By identifying high-risk patients with simple screening tools such as MNA and SARC-F, nutritional support and short-term prehabilitation programs can be planned before surgery. In addition, optimization of surgical timing and closer postoperative flap monitoring in these patients may facilitate early recognition of complications. This approach indicates that preoperative risk assessment for free flap surgery can serve not only a descriptive role but also function as a tool to guide clinical management. In the present study, free flap reconstructions performed at different anatomical sites were evaluated together. Complication patterns may also differ according to reconstructive site because of variations in tissue characteristics, mechanical stress, wound contamination, and local vascular conditions. Therefore, the association observed between functional nutritional frailty and postoperative complications should not be interpreted as indicating that nutritional status alone determines complication risk. Rather, functional nutritional frailty should be considered one component of a multifactorial process that also includes procedure-specific and anatomical factors. Because of the limited number of complication events within each anatomical subgroup, reliable site-specific complication rates could not be estimated, and formal subgroup analysis was not performed. This study has several limitations. First, its retrospective, single-center design may limit the generalizability of the results. However, the fact that all free flap procedures were performed by the same surgical team provided a more homogeneous patient population with respect to surgical technique and perioperative management. Second, inclusion of different reconstruction sites may introduce bias, since region-specific nutritional disturbances—such as dysphagia, tumor burden, or prior oncologic treatments—are more common in head-and-neck patients and could theoretically influence the relationship between nutritional status and complications. The higher rate of red blood cell transfusion in patients with functional nutritional frailty may reflect greater operative complexity, perioperative blood loss, pre-existing anemia, or reduced physiological reserve. Therefore, transfusion should be interpreted as a marker of overall patient and surgical burden rather than an isolated risk factor. Another limitation of this study is that perioperative antibiotic administration was individualized according to patient and surgical characteristics rather than following a standardized institutional protocol. This may have introduced treatment heterogeneity and could have influenced postoperative outcomes. Nonetheless, the primary aim of the study was to examine the impact of the patient's overall nutritional and functional reserve on free flap outcomes rather than to assess anatomical-site–specific nutritional abnormalities. Another limitation is that sarcopenia was assessed using clinical screening tools rather than imaging-based measurements of muscle mass. Nevertheless, this approach offers an important clinical advantage by demonstrating that nutrition-related functional frailty can be evaluated in routine practice using feasible, time-efficient methods. Another limitation of this study is that vascular factors such as the presence of atherosclerotic vessel disease and lipid profile parameters were not consistently available because of the retrospective study design. These variables may influence microvascular outcomes and should be considered in future prospective studies evaluating postoperative complications. Finally, the limited sample size and the relatively small number of flap-related complications necessitate validation of multivariate analysis results in larger patient cohorts. At the same time, inclusion of diverse reconstructive indications may enhance the generalizability of our findings by reflecting the broad spectrum of patients encountered in routine microsurgical practice.

**Table 5:** Comparison of low-risk and high-risk patients according to combined MNA/SARC-F screening.

Variable	Low risk (*n* = 51)	High risk (*n* = 29)	*p*-value
Age, years	50.9 ± 15.6	49.9 ± 14.5	0.678
BMI, kg/m²	27.1 ± 3.5	18.6 ± 3.6	<0.001
Hemoglobin, g/dL	13.66 ± 1.42	10.59 ± 1.59	<0.001
Total lymphocyte count	2.48 ± 0.77	1.10 ± 0.41	<0.001
Albumin, g/dL	4.19 ± 0.55	2.95 ± 0.93	<0.001
Prealbumin, g/dL	0.229 ± 0.033	0.158 ± 0.027	<0.001
Urea, mg/dL	20.3 ± 5.5	32.7 ± 4.6	<0.001
Creatinine, mg/dL	0.89 ± 0.14	1.48 ± 0.40	<0.001
Diabetes mellitus, *n* (%)	0 (0.0)	4 (13.8)	0.015
Active smoking, *n* (%)	9 (17.6)	6 (20.7)	0.771
Rehospitalization, *n* (%)	5 (9.8)	18 (62.1)	<0.001
Flap-related complication, *n* (%)	1 (1.9)	16 (55.2)	<0.001

Patients were classified as high risk if they had MNA-defined malnutrition/risk of malnutrition and/or SARC-F ≥4. Comparisons were performed using Mann–Whitney U test for continuous variables and Fisher’s exact test for categorical variables.

## Conclusion

The results of the present study suggest that functional nutritional frailty is associated with flap-related complications in patients undergoing free tissue transfer. Beyond classical biochemical nutritional parameters, incorporation of functional screening tools such as the MNA and SARC-F into preoperative assessment may aid early identification of high-risk patients. However, given the retrospective design, limited sample size, and small number of complication events, these findings should be interpreted with caution and require validation in larger prospective studies.

## Data Availability

The data supporting the findings of this study are available from the corresponding author upon reasonable request, subject to institutional and ethical restrictions.
